# Acquired antibiotic resistance of *Pseudomonas* spp., *Escherichia coli* and *Acinetobacter* spp. in the Western Balkans and Hungary with a One Health outlook

**DOI:** 10.3934/microbiol.2025020

**Published:** 2025-06-16

**Authors:** Chioma Lilian Ozoaduche, Katalin Posta, Balázs Libisch, Ferenc Olasz

**Affiliations:** 1 Agribiotechnology and Precision Breeding for Food Security National Laboratory, Department of Microbiology and Applied Biotechnology, Institute of Genetics and Biotechnology, Hungarian University of Agriculture and Life Sciences, 2100, Gödöllő, Hungary; 2 Doctoral School of Biology, Hungarian University of Agriculture and Life Sciences, 2100, Gödöllő, Hungary

**Keywords:** One Health, antibiotic resistance, Western Balkans, *P. aeruginosa*, *E. coli*, *A. baumannii*, high-risk clones, carbapenemase

## Abstract

An increasing rate of antibiotic resistance (AR) has been observed in the Gram-negative bacteria *A. baumannii*, *P. aeruginosa*, and *E. coli* in the human, environmental, and food animal domains worldwide, thus posing a serious global health challenge. Acquired AR genes of these species were overviewed from selected Western Balkans countries together with those from the European Union member states Croatia and Hungary. The AR determinants published from Albania, Bosnia-Herzegovina, Serbia, and Croatia included diverse acquired β-lactamase genes, with several of them possessing carbapenemase activity, such as *bla*_VIM_, *bla*_NDM_, *bla*_KPC_, *bla*_OXA-23_, *bla*_OXA-66_, and *bla*_OXA-72_. Furthermore, acquired aminoglycoside, chloramphenicol, fosfomycin, tetracycline, sulfonamide, quinolone, and/or colistin resistance determinants were detected in the three domains of the One Health approach. The *in vitro* AR profile of representative isolates have also been overviewed. Multidrug-resistant *P. aeruginosa* isolates of the ST235 high-risk clone were mainly reported within clinical settings. The distribution of the *E. coli* ST131 and *A. baumannii* ST2 high-risk clones in both clinical and environmental settings highlight their adaptability and effective dissemination. Systematic infection control practices are advised to combat the spread of antibiotic resistance, and further research from a One Health perspective is encouraged into its emergence and dissemination.

## 1. Introduction

Antibiotic resistance (AR) is an emerging global health challenge and one of the world's most serious threats today. Certain bacterial strains can acquire resistance to all (or nearly all) clinically used antibiotics, and Gram-negative bacteria make up the majority of the World Health Organization (WHO) priority list of antibiotic-resistant pathogens against which new treatments are needed [1]. AR is intimately linked to antibiotic usage, with drug abuse accelerating its emergence. Antibiotic abuse can take several forms, including unnecessary usage (as for example in non-bacterial diseases), excessive prescription (overuse), and poor antibiotic selection, dose, or duration [2].

The high levels of AR for several important bacterial species–antibiotic group combinations reported by the European Antimicrobial Resistance Surveillance Network (EARS-Net) [2] for 2020 showed that AR is a serious threat to public health, both in the European Union/European Economic Area (EU/EEA) and worldwide [3]. It has been estimated that if new novel drugs are not discovered or formulated, there could be no effective antibiotics available to treat these resistant pathogens by 2050 [4].

*Escherichia coli* is a major cause of bloodstream, intestinal, and urinary tract infections acquired in the community [5]. Antibiotics including ampicillin, amoxicillin/clavulanic acid, nitrofurantoin, fosfomycin, fluoroquinolones, cephalosporins, and trimethoprim/sulfamethoxazole have been found to cause high rates of resistance in uropathogenic *E. coli* strains [6]. *Pseudomonas* is a genus that is extensively found in both natural and aquatic habitats. Some of its species are opportunistic pathogens of humans and/or animals, while others can be harmful to plants [7]. These microbes can thrive in a wide range of environmental niches because of their metabolic versatility. Additionally, it has been well studied that the formation of biofilms in conjunction with antibiotic resistance may make it very challenging to eradicate *Pseudomonas* species from polluted environments or from illnesses in humans or animals [8]. Moreover, the presence of antibiotic-resistant *P. aeruginosa* has been found in wastewater treatment facilities [9]. The *Acinetobacter* species are known to cause a number of infections linked to healthcare [10], where *Acinetobacter* isolates from Southern and Eastern European countries, especially from the Balkans, can exhibit high rates of resistance to carbapenem antibiotics [10].

Based on a report by the WHO Regional Office for Europe/European Centre for Disease Prevention and Control, the most common underlying factors that contribute to the problem of non-prudent and excessive empirical prescribing of antibiotics in hospitals include the lack of appropriately applicable clinical guidelines or prescribing protocols, not sufficient diagnostics and diagnostic uncertainty, inappropriate physicians' knowledge and prescribing autonomy, and the influence of other factors [2]. Some developing countries continue to employ antibiotics for growth promotion to maintain the healthy state of animals, to increase productivity, and to raise incomes for the farmers [11].

Antibiotics have been widely utilized on dairy and other farms to prevent infections, where this kind of prophylaxis may be considered as a preventative group treatment for food animals [12]. However, Regulation (EU) 2019/6 of the European Parliament and of the Council on veterinary medicinal products (in force since 28 January 2022) states that antibiotic medicinal products should not be used for prophylaxis other than in exceptional cases and only for the administration to an individual animal [13]. On the one part, AR in bacteria may develop as a result of the usage of antibiotics in animals, with the potential to then spread to humans [14]. The overuse of antibiotics in poultry has been linked to a high degree of resistance in *E. coli* against therapeutically significant antibiotics such as penicillin, chloramphenicol, tetracycline, sulfonamides, and/or fluoroquinolones [15]. Additionally, airborne antibiotic-resistant bacteria and antibiotic resistance genes (ARGs) have been frequently detected on farms, and the abundance of some ARGs (such as *tet*, *sul*, *erm*, *bla*, *mec*, *aac*, *van*, *mcr* and *mdr*) in farm bioaerosols were reported [16].

Antibiotics or ARGs can reach the environment through urinary and fecal excretions from humans and domestic animals, through direct environmental contamination in aquaculture or plant production, and via waste streams from the production of antibiotics or from hospitals [17]. The spread of acquired resistance in bacterial populations can be caused by a vertical spread with resistant clones (that is, clonal dissemination), by the relocation of the ARG to a genetic element that can independently move between cells, and by the horizontal transfer of mobile genetic elements (MGEs) [17].

An increasing trend in the immigration into the Western Balkans region has been observed in the recent decade. Moreover, according to Eurostat, 23.8 million people (5.3%) of the 446.7 million people living in the EU on 1 January 2022 were non-EU citizens [18]. Tourism, the employment of non-EU citizens, and industrial and economic connections (e.g., Hungarian-owned-businesses in Western Balkans countries such as Croatia, Serbia) can also facilitate the dissemination of antimicrobial resistant bacterial strains and, in turn, affect public health in this region of Europe. The Western Balkans, known for their magnificent and diverse geographical regions, provide a great variety of habitats to support a wide range of natural and human impacted ecosystems [19]. Among these habitats, resistant bacteria of human and veterinary origins may be disseminated, in part, by migratory birds with contaminated food or water, and birds can also play a role in the ecology, circulation, and dissemination of antibiotic-resistant bacteria through their fecal depositions [20]–[22].

The aim of this review is to analyze and summarize vailable reports of antibiotic resistance determinants in selected counties of the Western Balkans region (Albania, Bosnia-Herzegovina, Serbia and Croatia) with a One-Health outlook, and to examine potential relationships with those from the neighboring EU member state Hungary.

## 2. Emergence of multidrug-resistant bacteria

Multidrug resistance refers to the ability of microorganisms, such as bacteria, viruses, fungi, and parasites, to resist the effects of multiple antimicrobial agents, where a bacterial isolate is considered multidrug-resistant (MDR) if the isolate is non-susceptible to at least one agent in ≥3 antimicrobial categories [23],[24]. Some of the mechanisms that might contribute to the MDR phenotype include genetic mutations which lead to the development of resistance to antibiotics, horizontal gene transfer, the exchange of genetic material between microorganisms, efflux pumps that can remove antibiotics from the cell, the appearance of novel enzymes or enzyme modifications that can degrade a particular antibiotic, biofilm development (i.e., complex communities of microorganisms adhered to surfaces and more resistant to antibiotics), and various mechanisms for the alterations of antibiotics [17],[24]. The *in vitro* AR profiles of representative isolates discussed in this review are available in Supplementary Table 1.

Some examples of high-risk MDR bacteria include extended-spectrum β-lactamase (ESBL)-producing *Enterobacteriaceae*, MDR *P. aeruginosa*, and carbapenem-resistant *Enterobacteriaceae* (CRE) [23],[24].

The population of the whole world, including the Western Balkans region, faces significant challenges of bacterial multidrug resistance [3],[23]. Studies have shown that the Western Balkans region also has a high prevalence of MDR bacteria [10],[25],[26]. Several factors can contribute to this high prevalence, including the overuse and misuse of antibiotics, a lack of effective infection control measures, insufficient surveillance and monitoring, and economic constraints and limited resources [2],[23],[24],[27].

Widespread elevated morbidity and mortality, financial hardships, and compromised antibiotic efficacy can be some of the outcomes of bacterial multidrug-resistance in the Western Balkans [3],[23]–[26]. The spread of ARGs in the human and animal populations increases the likelihood of disease transmission, reduces the efficacy of antibiotic treatments, and might also have serious economic consequences for animal husbandry, including a decreased output and higher costs [11],[12],[23],[24].

Additionally, the presence of these potent acquired (and thus transferable) ARGs in the environment has the potential to pollute water and soil, which puts human and animal health at risk. Thus, the widespread distribution of such ARGs can potentially have a substantial influence both on agricultural and natural ecosystems, including the disruption of microbial populations and a reduction of microbial biodiversity [11],[12],[23],[24].

### 2.1. Antibiotic resistance in the clinical setting in the Western Balkans

In Serbia, Lepsanovic et al. [28] first reported an ST235 *P. aeruginosa* clinical isolate that carried a *bla*_VIM-2-like_ metallo-β-lactamase gene. A PER-1 ESBL producing serotype O11 *P. aeruginosa* strain from a Serbian clinical setting also possessed an *aacA4* aminoglycoside acetyltransferase, *aadB* and *aadA2* aminoglycoside adenyltransferases, an *aphA* aminoglycoside phosphotransferase, and *bla*_OXA2_ resistance genes [29]. Jovcic and colleagues in 2011 and 2014 [30],[31] described the globally significant *bla*_NDM-1_ metallo-β-lactamase determinant in *P. aeruginosa* in Serbia. Moreover, studies by Kabic et al. revealed the occurrence of *bla*_NDM-1_, *bla*_GES-5_, *bla*_PER1_, *bla*_OXA-396_, and *bla*_OXA-488_ β-lactamases, *aadA6* aminoglycoside adenyltransferase, *aphA6*, *aph(3)-IIb*, *aph(6)Id*, and *aph(6)Ib* aminoglycoside phosphotransferases, and the *sul1* sulfonamide resistance gene in *P. aeruginosa* in Serbia [32]. Several potent ARGs were also identified in *A. baumannii* in Serbia by Kabic and coworkers [33]. The resistance genes *bla*_NDM-1_, *bla*_OXA-488_, *aac(6′)-Il*, *aph(3′)-IIb*, *ant(2″)-Ia*, *sul1*, *fosA*, and *catB7* were described in *P. aeruginosa* from Albania [34]. Additionally, acquired metallo-β-lactamases and other acquired resistance genes of *P. aeruginosa, E. coli*, and *A. baumannii* were also reported in the European Union (EU) member state Croatia, as summarized in Table 1 [28]–[44].

### 2.2. Antibiotic resistance in food animals in the Western Balkans

Acquired ARGs of *E. coli* have been characterized from some food animals in the Western Balkan [45], including *mcr-1*, *bla*_TEM-1B_, *bla*_CTX-M-1_, *aac(3)-IId*, *aph(30)-Ia*, *aadA5*, *sul2*, and *catA1* genes from pigs in Croatia (see Table 2).

**Table 1.** Acquired antimicrobial resistance genes in the clinical setting in the Western Balkans.

Location	Resistance genes	Sequence Type (Serotype)	References
	**Serbia**		
	***P. aeruginosa***		
*Belgrade*	*bla*_VIM-2-like_	ST235 (O11)	[28]
*Belgrade*	*bla*_PER-1_, *bla*_OXA-2_, *aacA4*, *aadA2*, *aadB*, *aphA*	ST235 (O11)	[29]
*Belgrade*	*bla*_NDM-1_	*N/A*	[30],[31]
*Belgrade*, *Kragujevac*, *Sombor*	*bla*_NDM-1_, *bla*_PER-1_, *bla*_GES-5_, *bla*_OXA-396_, *bla*_OXA-488_, *aadA6*, *aphA6*, *aph(3′)-IIb*, *aph(6′)Id*, *aph(6′)Ib*, *sul1*, *qac*	ST235, ST654	[32]
	***A. baumannii***		
*N/A*	*bla*_OXA-72_, *bla*_OXA-66_, *bla*_ADC-25_, *aadA2*, *aphA6*, *armA*, *tetB, sul1*, *sul2*, *strA*, *strB*, *dfrA12*	ST492	[35]
*Belgrade*, *Vojvodina*	*bla*_OXA-66_/*bla*_OXA-23_, *bla*_ADC-73_, *bla*_ADC-217_	ST2	[33]
*Belgrade*, *Vojvodina*	*bla*_NDM-1_, *bla*_OXA-72_, *bla*_ADC-30_, *aac(3′)-Ia*, *aadA*, *aadA2*, *aph(3′)-Ia*, *aph(3′)-VI*, *sul2*, *drfA1*, *dfrA12*, *catI*	ST492	[33]
*Belgrade*, *Vojvodina*	*bla*_OXA-72_, *bla*_ADC-74_, *aac(3)-Ia*, *aadA*, *aph(3′)-Ia*	ST636	[33]
*Belgrade*	*bla*_OXA-66_ */bla*_OXA-72_	ST636	[36]
	**Albania**		
	***P. aeruginosa***		
*N/A*	*bla*_NDM-1_, *bla*_OXA-488_, *bla*_PAO_, *aac(6′)-Il*, *aph(3′)-IIb*, *ant(2″)-Ia*, *sul1*, *fosA*, *catB7*, *crpP*	ST235	[34]
	*A. baumannii*		
*N/A*	*bla*_TEM-1_, *bla*_OXA-23_, *bla*_OXA-51_, *ampC*, *aph(3′)-Ia*, *aphA6*, *armA*, *tetB*, *sul2*, *strA*, *strB*	ST2/ST436	[37]
	**Croatia**		
	***P. aeruginosa***		
*Dalmatia*	*bla*_VIM-2_, *bla*_OXA-10_	ST235 (O11) and ST111 (O12)	[38]
*Zagreb*, *Dalmatia*	*bla*_VIM-1_, *bla*_VIM-2_, *bla*_PER-1_, *bla*_GES-7_	ST235 (O11), ST111 (O12)	[39]
	***E. coli***		
*Dubrovnik*, *Zagreb*, *Slavonski Brod*	*bla*_CTX-M-27_, *bla*_CTX-M-15_, *bla*_CTX-M-55_, *bla*_TEM-1_, *bla*_OXA-1_, *aadA2*, *aadA5*, *aac(6′)Ib-cr*, *aac(3)-IIa*, *tet(A)*, *sul1*, *sul2*, *strA*, *strB*, *fosA*, *catB3*, *dfrA12*, *mph(A)*	ST131	[40]
*Zagreb*	*bla*_CTX-M_, *bla*_OXA-48_	*N/A*	[41]
	***A. baumannii***		
*Osijek*	*bla*_OXA-23_, *bla*_OXA-66_, *bla*_ADC-25_, *aac(3)-Ia*, *aadA1*, *aph(3′)-VIa*, *aph(3″)-Ib*, *aph(6)-Id*, *armA*, *tet(B)*, *sul1*	*N/A*	[42]
*Zagreb*	*bla*_OXA-23_	*N/A*	[41]
	**Bosnia and Herzegovina**		
	***A. baumannii***		
*Mostar*	*bla*_OXA-23-like_, *bla*_OXA-40-like_, *bla*_OXA-51-like_, *bla*_OXA-69_, *bla*_OXA-72_, *bla*_ADC_, *aac(3)-Ia*, *aadA1*, *sul1*	ST642, ST636	[43]
	***E. coli***		
*Zenica-Doboj Canton*	*bla*_CTX-M-1_, *bla*_CTX-M-3_, *bla*_CTX-M-15_, *bla*_SHV-1_, *bla*_SHV-5_, *bla*_CMY-2_	*N/A*	[44]

**Table 2.** Acquired antimicrobial resistance genes in food animals in the Western Balkans.

Sample type (Location)	Resistance Genes	Sequence type (Serotype)	References
	**Croatia**	
	***E. coli***		
Pigs (*N/A*)	*bla*_CTX-M-1_, *bla*_TEM-1B_, *aac(3)-IId*, *aadA5*, *aph(3′)-Ia*, *sul2*, *mcr*-*1*, *catA1*	ST744	[45]

### 2.3. Antibiotic resistance in the environment in the Western Balkans

In Croatia, *bla*_TEM-116_ ESBL was detected in *Pseudomonas* spp. and in *E. coli* by Maravic et al. (2012) and Puljko et al. (2023) from coastal waters and hospital wastewater, respectively [46],[47]. Carbapenemase producing isolates were also described from wastewater treatment plant and dump site environmental samples [48]–[50]. Aminoglycoside acetyltransferase (*aac*), adenyltransferase (*aad*), phosphotransferase (*aph*) genes, and other types of ARGs were identified in *Acinetobacter* spp. by Higgins and coworkers [50] (Table 3). Cirkovic and colleagues identified antibiotic resistant *P. aeruginosa*, *E. coli*, and *A. baumannii* from wastewater in Belgrade [51]. Velhner *and* colleagues [52] reported *bla*_CTX-M-1_, *bla*_TEM-1_, *bla*_CMY-2_, *aadA1*, *tet(A)*, *tet(B)*, *sul1*, *sul2*, *sul3*, *strA*, *strB*, *cat1*, *dfrA1*, *dfrA7/17*, and *dfrA12* resistance determinants from black-headed gulls in Serbia.

**Table 3.** Acquired antimicrobial resistance genes in the environment in the Western Balkans.

Sample type (Location)	Resistance Genes	Sequence Type (Serotype)	References
	**Croatia**	
	***P. fluorescens***	
*Coastal waters (Kaštela)*	*bla*_TEM-116_	*N/A*	[46]
	***E. coli***	
*Hospital wastewater (Zagreb)*	*bla*_CTX-M-15_/*bla*_TEM-116_, *bla*_TEM-1_, *bla*_KPC-2_	ST131	[47]
	***A. baumannii***	
*Wastewater treatment plant (Zagreb)*	*bla*_OXA-23*-like*_, *bla*_OXA-40-like_, *bla*_OXA-51-like_	*N/A*	[48]
*Dump site (Rijeka)*	*bla*_OXA23_, *bla*_OXA72_	ST195, ST231	[49]
*Wastewater treatment plant (Zagreb)*	*bla*_OXA-23_, *bla*_OXA-66_, *aac(3)-Ia-like*, *aadA1*, *aph*(*3′)-VIa-like*, *armA*, *tet(B)-like*, *sul1*, *strA*, *strB*, *catA1-like*	ST195/ST2	[50]
	**Serbia**	
	***P. aeruginosa***	
*Wastewater (Belgrade)*	*bla*_PER-1_, *bla*_OXA-395_, *bla*_OXA-847_, *aph(3″)-Ib*, *aph(3′)-IIb*, *aph(3′)-VIb*, *aph(6)-Id*, *crpP*, *catB7*, *fosA*	ST348, ST2305	[51]
	***E. coli***	
*Black-headed gulls (Novi Sad)*	*bla*_CTX-M-1_, *bla*_TEM-1_, *bla*_CMY-2_, *aadA1*, *tet(A)*, *tet(B)*, *sul1*, *sul2*, *sul3*, *strA*, *strB*, *cat1*, *dfrA1*, *dfrA7/17*, *dfrA12*	ST38	[52]
*Wastewater (Belgrade)*	*bla*_NDM-1_, *bla*_OXA-1_, *bla*_SHV-12_, *bla*_TEM-1_, *bla*_OXA-10_, *bla*_OXA-48_, *aac(3)-IIe*, *aac(3)-IId*, *aac(3)-IIg*, *aac(6′)-Ib*, *aac(6′)-Ib-cr5*, *aac(6′)-Ib4*, *aac(6′)-IIc*, *aadA2*, *aph(3′)-Ia*, *aph(3′)-VI*, *aph(3″)-Ib*, *aph(6)-Id*, *tet(D)*, *tet(B)*, *sul1*, *sul2*, *catA1*, *catB3*, *dfrA12*, *dfrA14*, *qnrA6*, *catB3*, *cmlA5*	ST1133/ST1970, ST21/ST155, ST43/ST131	[51]
	***A. baumannii***	
*Wastewater (Belgrade)*	*bla*_OXA-23_, *bla*_OXA-66_, *bla*_OXA-72_, *aadA2*, *abaF*, *ant(3″)-IIa*, *aph(3″)-Ib*, *aph(3″)-Ib*, *aph(6)-Id*, *armA*, *tet(B)*, *sul1*, *sul2*, *dfrA12*	ST2/ST195, ST492/ST425	[51]

### 2.4. Antibiotic resistance in the clinical setting in Hungary

A great variety of antibiotic resistance genes have been identified in the clinical setting in Hungary, including ESBLs, and aminoglycoside, tetracycline, and sulfonamide resistance genes; see Table 4 for examples [53]–[62]. *bla*_VIM-4_ metallo-β-lactamase-producing *P. aeruginosa* clinical isolates characterized in Hungary included serotype O11 or O12 isolates in Budapest, Pécs, Mosonmagyaróvár, and other locations [53],[54]. The observations of recovering the same class 1 integron from different serotypes of *P. aeruginosa* from different locations indicated a role for a horizontal transfer in its dissemination and/or the repeated acquisition of this integron by various clinical strains [54]. *P. aeruginosa* clinical isolates that carried the aminoglycoside adenyltransferase genes *aadA13* and *aadB* with serotype O4 and ST175 were detected in Budapest, Gyula, Dombóvár, Veszprém, Balassagyarmat, Zalaegerszeg, and Szolnok, as well as an *aadB* determinant with serotype O6 and ST395 in Budapest, Miskolc, Debrecen, Székesfehérvár, and Pápa. This suggested that integrons may effectively contribute to the clonal dissemination of aminoglycoside resistance, which was likely due to the movement of infected or colonized individuals across different epidemiological settings [56]. Twelve human GenR *E. coli* strains and thirty-eight GenR *E. coli* strains of a food animal origin were examined and identified in Hungary with multidrug resistance, which led to the conclusion that the treatment of *E. coli* infections in humans and animals may be increasingly constrained by resistance genes in commensal and clinical strains [58]. Szmolka et al. (2012), Tóth et al. (2013), Nagy et al. (2023), and Gulyás et al. (2023) characterized *E. coli* clinical isolates that harbored *bla*_CTX-M-_type ESBL genes: *bla*_CTX-M-1_ [58]–[60] and *bla*_CTX-M-15_ [59]–[61]. The carbapenemase determinants *bla*_OXA-23_ and *bla*_OXA-72_ were identified in ST636 and ST492 *A. baumannii* [62].

### 2.5. Antibiotic resistance in the food animal setting in Hungary

*bla*_CTX-M_, *bla*_SHV_, and *bla*_TEM_-type β-lactamases have been identified in *E. coli* isolated from poultry, pigs, and cattle [58],[59]. Pigs and poultry were both shown to carry aminoglycoside adenyltransferases and tetracycline resistance genes *aadA* and *tet(A)* [63],[64]. Intestinal *E. coli* strains of food animals such as pigs, chickens, and red deer possessed aminoglycoside acetyltransferases, aminoglycoside phosphotransferases, β-lactamases, and tetracycline resistance genes [64]. Food producing animals such as domestic pigs and chickens harbored *bla*_TEM-1B_, *bla*_CMY-2_, *aac(3)-VIa*, *aadA1*, *tet(A)*, *tet(B)*, *tet(C)*, *sul1*, *sul2*, *strA*, and *strB* resistance genes [64],[65].

Acquired β-lactamases, aminoglycoside acetyltransferases, aminoglycoside adenyltransferases, aminoglycoside phosphotransferases, sulfonamide, and tetracycline resistance genes were repeatedly identified in other studies from food animals, as shown in Table 5 [58],[59],[63]–[66].

**Table 4.** Acquired antimicrobial resistance genes in the clinical setting in Hungary.

Hungarian clinical setting
Location	Resistance Genes	Sequence Type (Serotype)	References
	***P. aeruginosa***		
Pécs	*bla*_VIM-4_, *aacA4*	ST229 (O12)	[53],[54]
Győr	*bla*_VIM-4_, *bla*_OXA-2_, *aacA7*, *aacA8*	ST235 (O11)	[53],[54]
Budapest	*bla*_PER-1_, *bla*_OXA-2_, *bla*_OXA-74_, *aac(6′)-Ib-cr*, *cmlA7*	ST235 (O11)	[29]
Budapest	*bla*_VIM-2_, bla_VIM-4_, *aacA4*, *aacA7*	ST313 (O1), ST111 (O12), ST229 (O12)	[53],[54]
Budapest	*bla*_VIM-2_, *bla*_PER-1_	*N/A*	[55]
Gyula	*bla*_VIM-4_, *aacA4*	ST235 (O11)	[54]
Budapest	*aadA13*, *aadB*	ST175 (O4), ST395 (O6)	[56]
Gyula	*aadA13*, *aadB*	ST175 (O4)	[56]
Dombóvár	*aadA13*, *aadB*	ST175 (O4)	[56]
Veszprém	*aadA13*, *aadB*	ST175 (O4)	[56]
Balassagyarmat	*aadA13*, *aadB*	ST175 (O4)	[56]
Szolnok	*aadA13*, *aadB*	ST175 (O4)	[56]
Miskolc	*aadB*	ST395 (O6)	[56]
Debrecen	*aadB*	ST395 (O6)	[56]
Székesfehérvár	*aadB*	ST395 (O6)	[56]
Pápa	*aadB*	ST395 (O6)	[56]
6 diagnostic centres	*bla*_NDM_, *bla*_VIM_, *bla*_IMP_, *bla*_KPC_, *bla*_OXA-48-like_	*N/A*	[57]
	***E. coli***		
*N/A*	*bla*_CTX-M-1_, *bla*_SHV_, *bla*_TEM_, *bla*_OXA-1_, *aac(6′)-Ib*, *aadA1*-like, *aadA4*-like, *ant(2″)-Ia*, *tet(A)*, *tet(B)*, *sul1*, *sul2*, *strA*, *strB*, *catA1*, *catB3-like*, *floR*, *dfrA1*, *dfrA17*	*N/A*	[58]
*N/A*	*bla*_CTX-M-1_, *bla*_CTX-M-15_, *bla*_SHV-2_, *bla*_SHV-5_, *bla*_SHV-12_, *bla*_TEM-1_	ST131 (O25), (O15)	[59]
South-Pest	*bla*_CTX-M-1_, *bla*_CTX-M-15_, *acc(6′)-lb-cr*, *tet(A)*, *sul1*, *dfrA17*	ST43 (H4-O25)	[60]
Budapest	*bla*_NDM-5_, *bla*_CTX-M-15_, *bla*_TEM-1B_, *bla*_OXA-1_, *bla*_OXA-181_, *bla*_CMY-2_, *aac(6′)-Ib-cr*, *aadA2*, *aadA5*, *dfrA5*, *dfrA12*, *dfrA17*, *sul1*, *mdf(A)*, *mph(A)*, *erm(B)*, *catB3*, *tet(B)*, *qnrS13*	ST410	[61]
	***A. baumannii***		
*N/A*	*bla*_PER-1_	*N/A*	[55]
*N/A*	*bla*_OXA-23_, *bla*_OXA-72_	ST636, ST492	[62]

**Table 5.** Acquired antimicrobial resistance genes in the food/animal setting in Hungary.

	Hungarian food/animal setting		
Sample type (Location)	Resistance Genes	Sequence Type (Serotype)	References
	***E. coli***		
Poultry *(N/A)*	*bla*_TEM_, *aadA1*-like, *aadA2-*like, *aadA4*-like, *tet(A)*, *tet(B)*, *sul1*, *sul2*, *strA*, *strB*, *catA1*, *floR*, *dfrA1*, *dfrA12*, *dfrA17*, *dfrA19*	*N/A*	[58]
Pigs *(N/A)*	*bla*_TEM_, *aadA1*-like, *aadA2-*like, *aadA4*-like, *tet(A)*, *tet(B)*, *sul1*, *sul2*, *sul3*, *strA*, *strB*, *catA1*, *floR*, *cmlA1-*like, *dfrA12*, *dfrA14*, *dfrA17*, *dfrA19*, *dfrV*	*N/A*	[58]
Cattles *(N/A)*	*bla*_CTX-M-1_, *bla*_SHV_, *bla*_TEM_, *bla*_OXA-1_, *aadA1*-like, *aadA2-*like, *aadA4*-like, *tet(A)*, *tet(B)*, *sul1*, *sul2*, *sul3*, *strA*, *strB*, *catA1*, *floR*, *dfrA1*, *dfrA14*, *dfrA15*, *dfrA17*	*N/A*	[58]
Poultry, cattle or milk, pig *(N/A)*	*bla*_CTX-M-1_, *bla*_CTX-M-32_, *bla*_SHV-2_, *bla*_TEM-1_	(O162, O8)	[59]
Pigs (Pécs)	*aadA*, *tet(A)*, *strA*	(O141)	[63]
Domestic pig (Herceghalom)	*aadA1*, *tet(A)*, *tet(B)*	*N/A*	[64]
Chicken (Herceghalom)	*bla*_TEM-1B_, *bla*_CMY-2_, *aac(3)-VIa*, *aadA1*, *tet(A)*, *sul1*, *sul2*, *strA*, *strB*	*N/A*, *E. coli strain* K1G	[64]
Pigs (Herceghalom)	*tet(C)*	*N/A*	[65]
Duck *(N/A)*	*mcr-1*	ST162	[66]

### 2.6. Antibiotic resistance in environmental settings in Hungary

A great variety of ARGs were identified in the environmental setting in Hungary, as exemplified in Table 6 [61],[64],[67], including β-lactamases with a carbapenemase activity (such as *bla*_NDM-1_, *bla*_NDM-5_, *bla*_VIM-4_), aminoglycoside acetyltransferases, aminoglycoside adenyltransferases, aminoglycoside phosphotransferases, tetracycline, and sulfonamide resistance genes. The β-lactamase genes *bla*_NDM-1_, *bla*_VIM-4_, *bla*_SHV-12_, *bla*_TEM-1_, and *bla*_OXA-10_ were identified in the river Danube and black-headed gulls in Hungary [61]. Other determinants recovered from both the Danube and black-headed gulls included *aac(3′)-lld*, *aac(6′)-IIa*, *aac(6′)-Ib-cr*, *aadA1*, *aadA2*, *aadA5*, *aph(3′)-VI*, *aph(3″)-Ib*, and *aph(6)-Id* [61]. The *tet(A)*, *tet(B)*, and *sul1* genes were identified from both environmental sources (Table 6). *tet(B)*, *strA*, and *strB* resistance genes were detected from free-living red deer in Zsitfapuszta and Vörösalma in South-West Hungary [64]. The fallow deer and red deer samples were considered as environmental samples because these were free-living animals in their natural environmental habitat.

**Table 6.** Acquired antimicrobial resistance genes in the environmental setting in Hungary.

	Hungarian environmental setting		
Sample type (Location)	Resistance Genes	Sequence Type (Serotype)	References
	***E. coli***		
Danube (Budapest)	*bla*_NDM-1_, *bla*_VIM-4_, *bla*_SHV-12_, *bla*_TEM-1_, *bla*_OXA-10_, *bla*_CARB-12_, *aac(6′)-Ib-cr*, *aac(6′)-IIa*, *aadA1*, *aadA5*, *ant(2″)-Ia*, *aph(3′)-VI*, *aph(6)-Id*, *aph(3″)-Ib*, *tet(A)*, *sul1*, *sul2*, *catA1*, *floR*, *dfrA1*, *dfrA7*, *dfrA14*, *mdf(A)*, *mph(A)*, *mph(B)*	ST10	[61]
Danube (Budapest)	*bla*_NDM-1_, *bla*_CTX-M-24_, *bla*_TEM-1_, *bla*_OXA-9_, *bla*_OXA-10_, *aac(6′)-Ib-cr*, *aac(6′)-IIa*, *aadA1*, *aph(3′)-VI*, *ant(3″)-Ia*, *dfrA14*, *sul1*, *mdf(A)*, *mph(A)*, *erm(42)*	ST354	[61]
Danube (Budapest)	*bla*_NDM-5_, *bla*_CTX-M-15_, *bla*_TEM-1B_, *bla*_CMY-2_, *bla*_OXA-1_, *aac(3)-IId*, *aac(6′)-Ib-cr*, *aadA2*, *aadA5*, *aph(6)-Id*, *aph(3″)-Ib*, *tet(B)*, *sul1*, *sul2*, *catB3*, *dfrA12*, *dfrA17*, *mph(A)*, *mdf(A)*	ST410	[61]
Black-headed gulls (Budapest)	*bla*_NDM-1_, *bla*_VIM-4_, *bla*_TEM-1B_, *bla*_OXA-10_, *aac(3′)-lld*, *aac(6′)-IIa*, *aac(6′)-Ib-cr*, *aadA1*, *aadA2*, *aph(3′)-VI*, *aph(3′)-I*, *tet(A)*, *sul1*, *sul3*, *cmlA1*, *dfrA1*, *dfrA12*, *dfrA14*, *mdf(A)*, *erm(42)*, *mph(A)*	ST224	[61]
Black-headed gulls (Budapest)	*bla*_OXA-181_, *bla*_DHA-1_, *sul1*, *dfrA17*, *qnrB4*, *qnrS1*	ST372	[61]
Black-headed gulls (Budapest)	*bla*_NDM-1_, *bla*_SHV-12_, *bla*_TEM-1B_, *bla*_OXA-10_, *bla*_CMY-4_, *bla*_CMY-16_, *aac(3′)-lld*, *aadA1*, *aadA5*, *aph(3′)-VI*, *aph(3′)-Ia*, *aph(3″)-Ib*, *aph(6)-Id*, *rmtC*, *tet(A)*, *tet(B)*, *tet(D)*, *sul1*, *sul2*, *fosL1*, *cmlA1*, *catA1*, *floR*, *dfrA14*, *dfrA17*, *qnrA1*, *qnrB19*, *mdf(A)*, *mph(A)*	ST744	[61]
Fallow deer, red deer (Zsitfapuszta)	*tet(A)*, *tet(B)*, *strA*, *strB*		[64]
Red deer (Vörösalma)	*tet(B)*, *strA*, *strB*		[64]
Wild boar (Zemplén)	*acrD*	ST388 (O112ab:H2)	[67]

## 3. One Health outlook

MDR *P. aeruginosa* and *A. baumannii* isolates resistant to multiple agents can leave limited antimicrobial treatment options for clinicians. *P. aeruginosa* possesses several chromosomally encoded efflux pumps that can remove antimicrobial agents from the cell, thus reducing their effectiveness. At the same time, various acquired β-lactamases can be produced, such as VIM, IMP, and GES-type enzymes [68]. In general, MDR Gram-negative bacteria can produce a variety of acquired enzymes that modify aminoglycosides and other type of antibiotics, thus rendering them ineffective. Additionally, biofilm formation can protect these pathogens from antimicrobial agents and host immune responses [69]. Patients with cystic fibrosis or with compromised immune systems, such as those with HIV/AIDS or undergoing chemotherapy, are at an increased risk of developing MDR *P. aeruginosa* or other nosocomial infections [70].

MDR *E. coli* resistant to multiple antimicrobial agents can emerge by the production of β-lactamases, including CTX-M, SHV, and TEM-types, and by acquiring several other ARGs through a horizontal transfer [71]. Additionally, the spread of MDR *E. coli* can be facilitated besides other means by international travel and trade, which can introduce resistant strains into new regions by human and animal carriers. MDR *E. coli* can also cause community-onset infections such as urinary and intestinal tract infections [4]. MDR *A. baumannii* strains producing efflux pumps and/or various β-lactamases, such as OXA-23, OXA-24, and OXA-58 [72],[73], are often isolated from trauma patients, particularly those with combat-related injuries, and are a common cause of ventilator-associated pneumonia (VAP) in Intensive Care Units [73]. The WHO prioritized MDR *A. baumannii*, *P. aeruginosa*, and *Enterobacteriaceae* (e.g., *K. pneumonia*, *E. coli*) as critical cases of antibiotic-resistant bacteria. These Gram-negative pathogens can harbor potent carbapenemase-encoding genes, which enable the inactivation of most β-lactam antibiotics [74].

**Figure 1.**
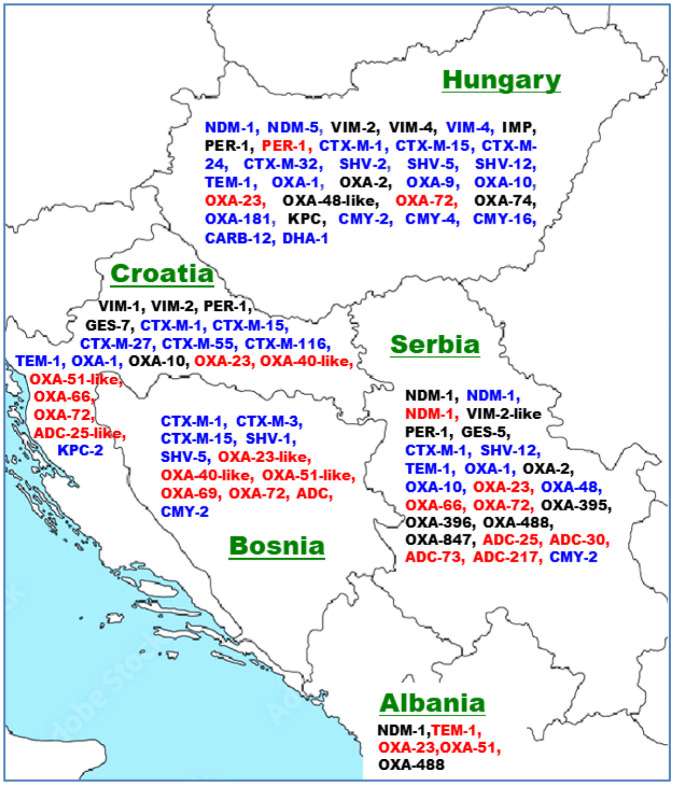
The distribution of various acquired β-lactamaseses in Hungary and in selected Western Balkans countries. Black, blue and red characters indicate enzymes reported for *P. aeruginosa*, *E. coli* and *A. baumanii* isolates, respectively.

The first report of the New Delhi metallo-β-lactamase (encoded by *bla*_NDM_), which confers resistance to a broad range of β-lactam antibiotics, was first published in 2009, where it was described in a *Klebsiella pneumoniae* isolate from a Swedish patient of Indian origin [75]. Subsequently, the *bla*_NDM-1_ gene was globally identified, including within the Balkans region. It was detected in clinical isolates of *P. aeruginosa*, *E. coli*, and *Acinetobacter* sp. in Serbia, a clinical *P. aeruginosa* isolate in Albania, and from *E. coli* isolates recovered from food animals and from environmental samples in Hungary (see Figure 1). Thus, this acquired carbapenemase has already been observed in several countries of this region of Europe and in all three domains of the One Health principles, thus highlighting the efficiency of its spread among Gram-negative bacteria.

*P. aeruginosa* isolates that belong to the sequence type 235 (ST235), an international high-risk clone that has the potential to cause nosocomial outbreaks with poor clinical outcomes, are a cause of serious concern; it is estimated that the ST235 sublineage emerged in Europe around 1984 and has successfully spread globally since then [34]. Metallo-β-lactamase-producing *P. aeruginosa* strains are linked to increased case fatality rates and invasive illnesses [54]. The *bla*_VIM_ metallo-β-lactamase genes encode another type of widespread carbapenemase enzyme, which was identified in clinical *P. aeruginosa* isolates in Serbia, Croatia, and Hungary. VIM-4-producing ST235 *P. aeruginosa* clinical isolates were reported from Budapest and Gyula in Hungary [53],[54], VIM-2-like producing clinical ST235 *P. aeruginosa* from Belgrade, Serbia [53],[54], and metallo-β-lactamase producing clinical ST235 *P. aeruginosa* isolates from Croatia [38] and Albania [34]. PER-1 ESBL positive clinical ST235 *P. aeruginosa* was reported from Budapest, Hungary. and Belgrade, Serbia [29]. These observations about the role of ST235 clinical isolates in disseminating high-risk antibiotic resistance determinants can be put into a wider context by considering their recovery from environmental samplings from hospital effluents/wastewaters in Germany and Brazil [76],[77]. Furthermore, ST235 *P. aeruginosa* strains were cultured from dogs and cats in Thailand, and carbapenem-resistant ST235 *P. aeruginosa* from dogs and cats in Japan [78],[79]. Overall, these findings highlight the necessity of a comprehensive One Health approach that includes samples from humans, animals, and their environment in uncovering possible routes of dissemination of such high-rick international clones of MDR Gram-negative bacteria.

Other resistance genes including acquired β-lactamases, aminoglycoside acetyltransferases, aminoglycoside adenyltransferases, aminoglycoside phosphotransferases, and sulfonamide resistance genes were identified in clinical, food animal, and environmental samples in the Western Balkans. The resistance gene *bla*_CMY-2_ identified in *E. coli* from free-living wild animals in Serbia was also identified in Hungary in *E. coli* strain K1G isolated from broiler chicken [64]. As shown in Tables 2 and 6, ST744 *E. coli* harbored *bla*_TEM-1B_, *aac(3′)-lld*, *aadA5*, *aph(3′)-Ia*, *sul2*, and *catA1* resistance genes in both pigs and gulls from Croatia and Hungary, respectively. Likewise, ST2 *A. baumannii* that carried the carbapenemase variants *bla*_OXA-23_ and *bla*_OXA-66_ was identified in a Serbian clinical setting, in Serbian wastewater, and in a Croatian wastewater treatment plant (Figure 1, Tables 1 and 3). From a One Health perspective, it should be highlighted that OXA-66- and OXA-72-coproducing ST2 *A. baumannii* was identified from an organic baby leaf mix purchased from a retail shop in Japan [80], which is similar to the ST2 *A. baumannii* found in Serbian wastewater [51] (Table 3). In addition, carbapenem-resistant ST2 *A. baumannii* with a *bla*_OXA23_-like gene was obtained from a two-year-old domestic cat in Pakistan [81], which is similar to clinical isolates from Serbia and Albania, and wastewater isolates from Croatia and Serbia (Tables 1 and 3). The most globally widespread high-risk clone and the most significant *Acinetobacter* species that infect humans is the ST2 carbapenem-resistant *A. baumannii* [51]. Additionally, it was noted that OXA-72-producing *A. baumannii* ST636 has been detected in clinical settings in Bosnia, Hungary, and Serbia (Tables 1 and 4).

Our findings highlight the detection of ST131 *E. coli* that harbors the β-lactamase variants *bla*_CTX-M-15_ and/or *bla*_TEM-1_ in the clinical setting of Croatia and Hungary, as well as in Croatian hospital wastewater and sewer outlets wastewater in Serbia (Figure 1, Tables 1, 3 and 4). ST131 *E. coli* which carried *bla*_CTX-M-15_ has also been reported from clinical samples of a Tanzanian tertiary hospital [82], diarrheic poultry in Tunisia [83], and from a Glaucous-winged gull in Russia [84]. A comprehensive multinational European investigation indicated that 6% of ESBL-producing *E. coli*, which was isolated from diverse companion animals, were classified as *E. coli* ST131, and the infrequent presence of *E. coli* ST131 supports the concept that humans, rather than companion or food-producing animals, are the principal reservoir of this high-risk clone [83]. ESBLs are predominant enzymes that can degrade cefotaxime, and the *bla*_CTX-M-15_ variant is considered to be globally dominant [82]. ST131-O25b-B2 *E. coli* is a prominent cause of serious human extraintestinal infections, particularly community-acquired urinary tract infections; it has also been observed in companion and non-companion animals that have had human contacts [83]. CTX-M-15-producing ST131 *E. coli* detected in wild birds was proposed to be of a human origin, with a potential of clonal dissemination even into environments lacking antibiotic pressure [84].

Our review underscores the significant contribution of high-risk clones to the global spread of antibiotic resistance. Notably, ST235 in *P. aeruginosa*, ST131 in *E. coli*, and ST2 in *A. baumannii* have emerged as prominent examples of high-risk global clones, exhibiting a widespread distribution in clinical settings, wastewater, and animals, thereby emphasizing the need for targeted surveillance and intervention strategies.

Based on the main findings of this literature review, a high variety of ESBLs and carbapenem resistance mechanisms were demonstrated in bacteria identified from the Western Balkans countries, alongside strains from Hungary (Tables 4–7). The dissemination of multidrug-resistant Gram-negative pathogens is considered a serious public health issue, both in this region of Europe as well as globally [85]. The One Health approach recognizes the interconnection and mutual influence of its three domains, and their interconnections create pathways by the clonal transmission of bacteria and by mobile genetic elements (MGEs) [74],[86]–[89].

**Table 7.** ARGs in Hungary and Western Balkans countries of study.

AR Class	Bacterial Strain	Country	AR genes
Quinolone	*E. coli*	Serbia	*qnrA6*
		Hungary	*qnrS13*, *qnrB4*, *qnrA1*, *qnrB19*, *qnrS1*
Tetracycline	*E. coli*	Hungary	*tetA*, *tetB*, *tetD*
		Serbia	*tetA*, *tetB*, *tetD*
		Croatia	*tetA*
	*A. baumannii*	Serbia	*tetB*
		Albania	*tetB*
		Croatia	*tetB*
Phenicol	*P. aeruginosa*	Hungary	*cmlA7*
		Serbia	*catB7*
		Albania	*catB7*
	*E. coli*	Hungary	*catA1*, *catB3*, *floR*, *cmlA1*
		Serbia	*catA1*, *catB3*, *cmlA5*
		Croatia	*catA1*, *catB3*
	*A. baumannii*	Serbia	*catI*
		Croatia	*catA1*
Aminoglycoside	*P. aeruginosa*	Hungary	*aacA4*, *aacA7*, *aacA8*, *aac(6′)-Ib-cr*, *aadA13*, *aadB*
		Serbia	*aacA4*, *aadA2*, *aadB*, *aadA6*, *aphA6*, *aph(3′)-IIb*, *aph(6′)Id*, *aph(6′)Ib*, *aph(3″)-Ib*, *aph(3′)-IIb*, *aph(3′)-VIb*, *aph(6)-Id*
		Albania	*aac(6′)-Il*, *aph(3′)-IIb*, *ant(2″)-Ia*, *aph(3′)-Ia*, *aphA6*, *armA*, *strA*, *strB*
	*E. coli*	Hungary	*aac(6′)-Ib*, *ant(2″)-Ia*, *aac(3)-lld*, *aac(6′)-Ia*, *aac(6′)-Ib-cr*, *aadA1*, *aadA2*, *aadA4-like*, *aadA5*, *aph(3′)-VI*, *aph(3′)-I*, *aac(3)-VIa*, *aadA1*, *aph(3″)-Ib*, *aph(6)-Id*, *aac(3)-Id*, *aac(6′)-IIa*, *strB*, *strA*
		Serbia	*aadA1*, *aac(3)-IIe*, *aac(3)-IId*, *aac(3)-IIg*, *aac(6′)-Ib*, *aac(6′)-Ib-cr5*, *aac(6′)-Ib4*, *aac(6′)-IIc*, *aadA2*, *aph(3′)-Ia*, *aph(3′)-VI*, *aph(3″)-Ib*, *aph(6)-Id*, *strA*, *strB*
		Croatia	*aadA2*, *aadA5*, *aac(3)-IId*, *aadA5*, *aph(3′)-Ia*, *aadA2*, *aac(6′)Ib-cr*, *aac(3)-IIa*, *strA*, *strB*
	*A. baumannii*	Serbia	*aadA2*, *aphA6*, *aac(3′)-Ia*, *aph(3′)-Ia*, *aph(3′)-VI*, *aac(3)-Ia, aadA*, *aph(3′)-Ia*, *abaF*, *ant(3″)-IIa*, *aph(3″)-Ib*, *aph(6)-Id*, *armA*, *strA*, *strB*
		Croatia	*aadA1*, *aac(3)-Ia*, *aph(3′)-VIa*, *aph(3″)-Ib*, *aph(6)-Id*, *aph(3′)-Ia*, *aac(3)-IId*, *aadA5*, *aac(3)-Ia-like*, *aph(3′)-VIa-like*, *armA*, *strA*, *strB*
		Bosnia and Herzegovina	*aac(3)-Ia*, *aadA1*
Macrolide	*E. coli*	Hungary	*ermB, erm42*
Sulphonamide	*P. aeruginosa*	Serbia	*sul1*
		Albania	*sul1*
	*A. baumannii*	Serbia	*sul1*, *sul2*
		Albania	*sul2*
		Croatia	*sul1*
		Bosnia and Herzegovina	*su1*
	*E. coli*	Serbia	*sul1*, *sul2*, *sul3*
		Croatia	*sul1*, *sul2*
		Hungary	*sul1*, *sul2*, *sul3*
Colistin	*E. coli*	Croatia	*mcr-1*
		Hungary	*mcr-1*
Trimethoprim	*A. baumannii*	Serbia	*dfrA12*
	*E. coli*	Serbia	*dfrA1*, *dfrA7/17*, *dfrA12*, *dfrA14*
		Hungary	*dfrA1*, *dfrA5*, *dfrA12*, *dfrA14*, *dfrA15*, *dfrA17*, *dfrA19*, *dfrV*
		Croatia	*dfrA12*
β-lactam	*P. aeruginosa*	Hungary	*bla*_VIM-4_, *bla*_OXA-2_, *bla*_VIM-2_, *bla*_PER-1_, *bla*_OXA-74_, *bla*_OXA-48-like_, *bla*_NDM_, *bla*_VIM_, *bla*_IMP_, *bla*_KPC_
		Serbia	*bla*_NDM-1_, *bla*_VIM-2-like_, *bla*_PER-1_, *bla*_OXA2_, *bla*_GES-5_, *bla*_OXA-396_, *bla*_OXA-488_, *bla*_OXA-395_, *bla*_OXA-847_
		Croatia	*bla*_VIM-2_, *bla*_OXA-10_, *bla*_VIM-1_, *bla*_VIM-2_, *bla*_PER-1_, *bla*_GES-7_
		Albania	*bla*_NDM-1_, *bla*_OXA-488_, *bla*_PAO_
	*E. coli*	Hungary	*bla*_CTX-M-1_, *bla*_OXA-1_, *bla*_CTX-M-15_, *bla*_OXA-181_, *bla*_CTX-M-32_, *bla*_SHV-2_, *bla*_TEM-1_, *bla*_VIM-4_, *bla*_SHV-5_, *bla*_SHV-12_, *bla*_DHA-*1*_, *bla*_CMY-2_, *bla*_CMY-4_, *bla*_CMY-16_, *bla*_NDM-1_, *bla*_NDM-5_, *bla*_CTX-M-24_, *bla*_OXA-9_, *bla*_OXA-10_, *bla*_CARB-12_
		Serbia	*bla*_CTX-M-1_, *bla*_CMY-2_, *bla*_NDM-1_, *bla*_OXA-1_, *bla*_SHV-12_, *bla*_TEM-1_, *bla*_OXA-10_, *bla*_OXA-48_
		Croatia	*bla*_CTX-M-27_, *bla*_TEM-1_, *bla*_OXA-1_, *bla*_OXA-48_, *bla*_CTX-M-1_, *bla*_CTX-M-15_/*bla*_TEM-116_, *bla*_CTX-M-15_, *bla*_CTX-M-55_, *bla*_KPC-2_
		Bosnia and Herzegovina	*bla*_CTX-M-1_, *bla*_CTX-M-3_, *bla*_CTX-M-15_, *bla*_SHV-1_, *bla*_SHV-5_, *bla*_CMY-2_
	*A. baumannii*	Hungary	*bla*_PER-1_, *bla*_OXA-23_, *bla*_OXA-72_
		Serbia	*bla*_OXA-72_, *bla*_OXA-66_, *bla*_ADC-25_, *bla*_OXA-23_, *bla*_ADC-73_, *bla*_ADC-217_, *bla*_NDM-1_, *bla*_ADC-30_, *bla*_ADC-74_
		Croatia	*bla*_OXA-23_, *bla*_OXA-66_, *bla*_ADC-25_, *bla*_OXA-40-like_, *bla*_OXA-51_-_like_, *bla*_OXA-72_
		Albania	*bla*_TEM-1_, *bla*_OXA-23_, *bla*_OXA-51_
		Bosnia and Herzegovina	*bla*_OXA-23-like_, *bla*_OXA-40-like_, *bla*_OXA-51-like_, *bla*_OXA-69_, *bla*_OXA-72_, *bla*_ADC_

**Figure 2.**
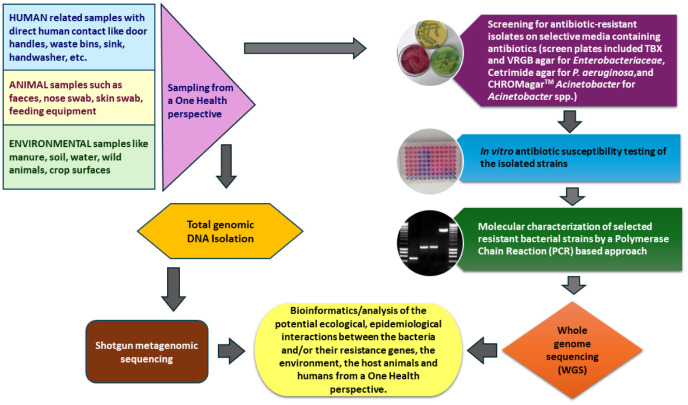
A graphical presentation on screening for antibiotic resistant bacteria in the Agribiotechnology and Precision Breeding for Food Security National Laboratory in Hungary [64],[65],[67],[87],[89].

A flowchart summarizing possible sample collection and analysis steps for molecular microbiological studies based on a One Health approach is provided in Figure 2. Some potential measures that can be considered to reduce the emergence and dissemination of antimicrobial resistance between the three One Health domains may include implementing or improving the sewage systems and wastewater treatment plants, reducing the amount of antibiotics consumed by humans and animals through market regulation, favoring more labile antibiotics, controlling pharmaceutical effluents, reducing the veterinary use of antibiotics, improving hygiene, and regulating the use of antibiotics, according to the proposal of Martak and colleagues [88].

It has been disclosed that the average Global One Health Index - Antimicrobial Resistance (GOHI-AMR) score for 146 nations is 39.85 [90]. A publication by Lancet in 2024 estimated that bacterial AMR was responsible for 4.71 million (95% uncertainty intervals 4.23–5.19) deaths in 2021, including 1.14 million (1.00–1.28) deaths attributable to bacterial AMR. The AMR burden is expected to rise to 1.91 million attributable deaths and 8.22 million associated deaths in 2050, with sub-Saharan Africa and south Asia bearing the brunt of this increase in absolute numbers [91].

## 4. Conclusions

The Western Balkans region together with their neighboring EU countries face significant challenges in addressing the growing issue of bacterial multidrug resistance. To combat this threat, it is essential to adopt a multifaceted approach that includes improving antibiotic stewardship, enhancing infection control measures, strengthening surveillance and monitoring, investing in research and development, and fostering regional collaborations. Consequently, it is also advised that a One Health approach shall be considered and followed during such efforts, which is similar to the principles of the Agribiotechnology and Precision Breeding for Food Security National Laboratory in Hungary for the screening protocols of antibiotic-resistant bacteria. Further research is needed to discover novel antimicrobial agents and alternative antimicrobial treatments and regional collaborations should be fostered to address the global threat of multidrug-resistant pathogens.

## Use of AI tools declaration

The authors declare they have not used Artificial Intelligence (AI) tools in the creation of this article.


